# Preparation of starch/PVA/Cu-NPs bio-nanocomposites in packing as preservation of UF soft cheese

**DOI:** 10.1038/s41598-026-44328-4

**Published:** 2026-04-04

**Authors:** Heba A. El-Refai, Sanaa K. Gomaa, Rania A. Zaki, Samah M. El-Sayed, Hoda S. El-Sayed, Youssef R. Hassan, Abdelmonem Elrefaey, M. E. Abd El-Aziz

**Affiliations:** 1 Chemistry of Natural and Microbial Products Department, National Research Centre, 33 El Bohouth St. (former El Tahrir St.), Dokki, Giza, P.O. 12622, Egypt; 2 Dairy Science Department, National Research Centre, 33 El Bohouth St. (former El Tahrir st.), Dokki, P.O. 12622, Giza, Egypt; 3 Packaging Materials Department, National Research Centre, 33 El Bohouth St. (former El Tahrir st.), Dokki, Giza, P.O. 12622, Egypt; 4 School of Computing and Augmented Intelligence, Arizona State University, Tempe, Arizona, AZ 85281 USA; 5 Polymers and Pigments Department, National Research Centre, 33 El Bohouth St., Dokki, Giza, P.O. 12622, Egypt

**Keywords:** Biosynthesized CuO-NPs, St/PVA bio-nanocomposites, Soft cheese, Active food packaging, Edible coatings, Barrier and antimicrobial properties, Biotechnology, Chemistry, Materials science, Microbiology, Nanoscience and technology

## Abstract

This study represents the biosynthesis of copper oxide nanoparticles (CuO-NPs) via bacteria and their utilization in the synthesis of a bio-nanocomposite based on starch (St) and polyvinyl alcohol (PVA) to be applied in preserving ultrafiltration (UF) soft cheese. The bio-nanocomposites were synthesized by blending St/PVA with a ratio of 4:1 wt, wt and loaded with various concentrations of CuO-NPs (0, 20, 40, and 80 mg) to obtain bio-nanocomposite coatings S1, S2, S3, and S4, respectively. The coatings’ mechanical strength, water and oxygen barrier quality, morphology, and crystallinity were all studied. The chemical composition, microbial load, total solids, and pH changes for uncoated and coated cheese samples that were stored at 5 ± 2 °C for 60 days were evaluated. Results showed that films loaded with CuO-NPs significantly improved the mechanical and barrier properties of the prepared films, in addition to reducing the moisture loss and microbial growth and extending the shelf life of the coated cheese. In addition, the bio-nanocomposite coating loaded with higher CuO-NP concentrations (S3 and S4) showed superior antimicrobial effects, indicating their potential as active edible coatings for cheese preservation.

## Introduction

Chemical synthesis is widely regarded as hazardous, costly, and environmentally unfriendly^1^. Eco-friendly green chemistry now supports the biological production of materials. This has led to nanobiotechnology, which uses organisms like bacteria, fungi, and plants in various chemical and physical processes^2^. Among various microorganisms, bacteria are considered ideal for nanoparticle (NPs) synthesis due to their notable advantages, including high stability, rapid growth, simple cultivation, tolerance to toxic heavy metals, and the ability to sustainably produce NPs on a large scale under mild conditions^3,4^. CuO-NPs have attracted significant interest due to their unique properties, including electrical, magnetic, thermal, and antimicrobial characteristics. With optical and catalytic features, they are utilized in electronics, energy devices, sensing technologies, thermal management, and the production of cosmetics and pharmaceuticals^5,6^. Design of Experiments (DOE) provides a structured framework for efficiently identifying which process factors have a significant impact on key outcomes. This report presents a comprehensive analysis of a 2^4−1^ fractional factorial screening experiment. The primary goal was to screen four factors, salt conc., filtrate, temp., and pH, to determine their influence on two critical responses reflecting NPs production: weight and optical density (O.D.). The specific objectives were to provide clear, data-driven recommendations for process control and future optimization.

In addition to enhancing food’s quality, safety, and longevity, the packaging process also enhances its sensory qualities^7,8^. Indeed, petroleum-based products are usually used in packaging^9^. The global environment is facing significant issues today. Materials may, for instance, build up in the environment and take hundreds of years to break down, and they are energy-intensive. As a result, experts from all over the world are now concentrating on finding solutions to environmental protection problems as well as reducing expenses and energy needs. Applying biodegradable polymers from natural sources that easily break down into less dangerous compounds is one viable alternative^10–12^.

A soluble starch is a renewable, compatible, and biodegradable biopolymer that can form films with other biopolymers, making it valuable in packaging applications^13^. Soluble starch-based films provide an eco-friendly alternative to synthetic polymers, offering good oxygen barrier properties and moderate mechanical strength. To improve flexibility, durability, and moisture resistance, it is frequently reinforced with nanoparticles or combined with plasticizers^14,15^. This makes it appropriate for use in food packaging and other environmentally friendly applications^16,17^. However, polyvinyl alcohol (PVA) is a petroleum-based polymer. It is one of the most important polymers with a wide range of industrial uses and is authorized for use in food contact applications^18,19^. It is the only synthetic polymer that is fully biodegradable, has outstanding mechanical properties, and exhibits good water solubility and high polarity^7,20^. Certain metal oxides have strong antibacterial qualities when they are in nanoform. By interacting with cellular membranes, producing reactive oxygen species, breaking down protein structures, and interfering with enzymatic and DNA replication activities, they prevent the growth of bacteria.

This work aims to use CuO-NPs bio-synthesized by *Bacillus safensis* FO-36b into starch-PVA bio-nanocomposites for the preservation of UF soft cheese. Bacterial biosynthesis produces uniformly tiny, biocompatible NPs with natural capping agents that improve dispersion, stability, and food safety, in contrast to chemically manufactured NPs that need hazardous reagents and significant energy. These NPs, which were optimized by fractional factorial design, increase the bio-nanocomposite’s mechanical strength, barrier qualities, and antibacterial efficacy. This prolongs the shelf life of cheese and addresses plastic pollution by using completely biodegradable packaging.

## Materials and methods

### Materials

Soluble starch (St; Botanical source: Potato, CAS NO.: 9005-84-9, amylose content 28%, ACS grade) was obtained from Oxford Lab Fine Chem LLP, India (MW = 342.30 g/mol). Polyvinyl alcohol (PVA; M.W.= 30000, degree of hydrolysis = 98–99%), copper sulfate pentahydrate (98%, CuSO_4_.5H_2_O), and nutrient agar were purchased from Sigma-Aldrich. Milk retentate was obtained from the Dairy Industry Unit, Animals Production Research Institute, Ministry of Agriculture, Giza, Egypt. Microbial rennet powder (RENIPLUS) extracted from *Mucor miehei* was purchased from Gaglio Star, Spain. While a nearby market provided salt. Other chemicals were of analytical grade.

### Screening bacteria for the production of CuO-NPs

Ten bacterial isolates were evaluated for their potential to biosynthesize CuO-NPs. Each isolate was cultured in 100 mL nutrient broth, incubated at 35 °C for 48 h in a rotary shaker at 120 rpm. Following incubation, the cultures were centrifuged at 10,000 rpm for 10 min using 50 mL Falcon tubes to separate the biomass. The resulting cell-free supernatants were employed as bioreductants for the green synthesis of CuO-NPs.

### Biosynthesis of CuO-NPs

The biosynthesis of CuO-NPs was carried out following the method described by Nassar et al^21^., with slight modifications. A volume of 13 mL of the bacterial culture filtrate was gradually added, under continuous stirring, to a reaction vessel containing 2 mM CuSO_4_−5H_2_O dissolved in 5 mL of deionized water. The reaction mixture was heated to 60 °C, and the pH was adjusted to the pH was adjusted to 10 using 0.1 M NaOH. The mixture was then left undisturbed overnight to allow NPs formation of NPs. After incubation, the supernatant was carefully removed, and the resulting precipitate (CuO-NPs) was collected and washed with deionized water by centrifugation at 5000 rpm for 10 min. This washing process was repeated three times through successive precipitation and re-dispersion in deionized water, followed by a final rinse with absolute ethanol. The purified NPs were then dried at 60 °C and stored for subsequent analysis.

### Optimization of the biosynthesized CuO-NPs

#### Design selection: fractional factorial

To improve efficiency over a 16-run full factorial, a 2^4−1^ one-half fractional factorial design was selected, requiring only 8 factorial runs and 2 center points. This reduces experimental cost while allowing for estimation of all main effects. Four parameters (CuSO_4_ (Mm), pH, temperature (°C), and filtrate concentration (ml)) in 10 combinations arranged in accordance with the design (Table 1).

**Table 1:** Experimental Design and Rationale.

Factors	-	0	+
CuSO_4_ (Mm) (x_1_)	1	2	3
Filtrate (ml) (x_2_)	10	13	16
Temperature (°C) (x_3_)	40	60	80
pH (x_4_)	8	10	12

### Preparation of St/PVA/CuO-NPs bio-nanocomposite

Starch was continuously stirred in water to prepare solution 4% at room temperature. After that, the solution was cooked at 80 °C for 30 min to get solution (A). Polyvinyl alcohol was dissolved in hot water at 90 °C for 30 min to get solution 2% to get solution (B). To prepare the St/PVA/CuO-NPs bio-nanocomposite, the blend of St/PVA was prepared first by mixing the previous solutions A: B under stirring for 30 min at 80 °C with a ratio of 4:1 (wt: wt) after different weights of CuO-NPs (0, 20, 40, and 80 mg) were added to obtain bio-nanocomposite coatings S1, S2, S3, and S4, respectively.

### Characterization

After the preparation of the bio-nanocomposite coatings solutions S1, S2, S3, and S4, the mixtures were poured onto clean Teflon plates and allowed to dry at room temperature (25 ± 2 °C) for 48 h, and then further dried in a vacuum oven at 40 °C for 24 h to remove residual moisture. The resulting films were used for the following characterization.

The morphology of bio-nanocomposites was characterized by scanning electron microscopy with a dispersive energy spectrometer (SEM-EDX; JSM 6360LV, JEOL/Noran, USA). Before analysis, samples were placed as far away from the target as possible to avoid damage, sputter-coated with a thin layer of gold at a low deposition rate, and then chilled. The images were created using an accelerating voltage of 20 kV.

Crystallographic properties of as-prepared bio-nanocomposites were examined via X-ray diffraction (XRD) using a Shimadzu 7000 diffractometer (Japan), operating in the 2θ range of 2–80° with a step size of 0.02° and utilizing CuKα radiation (λ = 1.5418 Å).

The mechanical properties of bio-nanocomposite film were evaluated using an LK10k universal testing machine (Hants, UK) equipped with a 1 kN load cell, operating at a speed of 5 mm.min^[-1^. Mechanical properties were tested according to ASTM D638-91, using the average of three replicates per sample to determine the tensile strength (MPa) and elongation (mm).

The water vapor transmission rate (WVTR) was measured using the cup method with a Water Vapor Permeability Analyzer (GBI W303 (B), China). The WVTR was determined based on the amount of water vapor passing through a unit area of the film per unit time under controlled conditions of 38 °C temperature and 4% humidity, following the ASTM E96 standard.

Oxygen transmission rate (OTR) was measured with an N530-B Gas Permeability Analyzer, China, according to ASTM D1434-82 (2003) and ISO2556-2001.

### Antibacterial activity of CuO-NPs and bio-nanocomposite film

Antibacterial activity of CuO-NPs was assessed against the Gram-negative bacteria (*Escherichia coli* NRRL B210) and Gram-positive bacteria (*Bacillus cereus ATCC 6629*) by the well diffusion agar method. The optical density of the inoculum was adjusted to 0.125 for freshly grown bacteria, and wells were made to add CuO-NPs at various concentrations (10, 20, 30, and 40 mg/ml). Pieces of the different composite films concentration (1,2, and 3) (10 × 10mm^2^) were placed on the inoculated agar plate surfaces and kept at 37 °C for overnight inhibition zone surrounding the samples was determined, and ceftriaxone was used as an antimicrobial reference for bacteria. The plates were incubated for 24 h at 37 °C. Antimicrobial activities (positive control) were evaluated by measuring the inhibition zone diameter (cm) while the negative control showed no inhibitory effect^22^.

### Manufacturing of Ultrafiltration soft cheese

Freshly whole cream UF retentate was used to make ultrafiltration soft cheese. It was treated at 80 °C for five minutes, salted with 1.5% salt, cooled, and adjusted to 42 °C^23^. Microbial rennet powders of 3% were added to UF retentate, and the cups were then packed in 50 mL plastic cups and allowed to coagulate for approximately two hours at 42 °C. The resulting ultrafiltration soft cheese was moved to the fridge to harden up. Five groups of the UF-retentate were produced; the first, without any addition, served as an uncoated control. The second was coated with a St/PVA blend (S1). The third, fourth, and fifth groups were coated with as-prepared coatings S2, S3, and S4, respectively. The covered cheese treatments were kept in the refrigerator at 5 ± 2 °C for 60 days, and samples were analyzed when they were still fresh and every 15 days. All determinations were done in triplicate.

### Cheese chemical analysis

The contents of UF- soft cheese in terms of total solids, total nitrogen, fat, and ash were ascertained using the Association Official Analytical Chemist^24^. Multiplying the TN % by 6.38 yielded the protein content. A pH-meter with microprocessor (Hanna Instruments Model 170300, Ingold, Knick, Germany) was used for measuring the pH value. The carbohydrate percentage is calculated from [100 – (Moisture + Ash + Protein + Fat)].

### Microbiological analysis of cheese samples

Aseptically, 25 g of various coated and uncoated cheese samples were added to 225 mL of buffered peptone water (0.1% w/v), and they were well mixed for one minute at room temperature. After making serial decimal dilutions using saline (0.9% w/v), 1 mL of the corresponding dilutions was distributed or poured onto agar plates. Once incubated at 35 °C for 48 h, the total number of bacteria was counted using plate count agar medium (Oxide). Spore-forming bacterial counts were found by heating the initial dilution of samples to 80 °C for 10 min, quickly cooling them with plates containing plate count agar medium (Oxoid), and then incubating them at 35 °C for 48 h. Coliform counts were identified using the Violet Red Bile Agar Medium (Oxide), which was incubated for 24 h at 35 °C. Mold and yeast counts were determined using the chloramphenicol rose Bengal agar medium (Oxide) and an incubation period of four days at 25 °C. The American Public Health Association’s standard procedure^25^ was followed for all microbiological analyses, and log CFU/g counts were carried out in triplicate.

### Statistical evaluation

The GLM technique was used for statistical analysis utilizing SAS^26^ software. The means were compared using analysis of variance (ANOVA) and Duncan’s multiple comparison approach. All experiments in this study were done in triplicate, and the results were averaged. The significance of differences was set at *p* < 0.05.

## Results and Discussion

### **Screening of bacterial isolates for CuO-NPs synthesis**

This experiment aims to select strains capable of reducing CuSO4 for CuO-NPs that was evidenced by a visible color change from blue to black after the addition of 2 mM aqueous CuSO4 to the bacterial cell-free filtrate. *Bacillus Safensis* FO-36b (Gen Bank accession number NR_113945.1) was isolated from soil located in upper Egypt was selected to synthesize CuO-NPs. Proteins and secondary metabolites with hydroxyl, carbonyl, and amine functional groups are among the enzymatic and non-enzymatic biomolecules found in the bacterial filtrate that are responsible for the reduction of Cu2 + ions. A strong UV-Vis absorption peak at 300 nm indicated the formation of CuO-NPs. The absorption peaks that were found matched those that Alali et al^27^. described, supporting the successful formation of CuO-NPs. The ability of Bacillus species to produce NPs is well-known. However, in the forefront of nanotechnological research is the use of Bacillus species such as *B. licheniformis* and *B. subtilis*,* which* have also been reported to synthesize CuO nanoparticles that can reliably carry out the biosynthesis of NPs with specific attributes, like having a particular composition and size^28^.

### Optimization of CuO-NPs (exploratory data analysis (EDA))

**Fig. 1:**
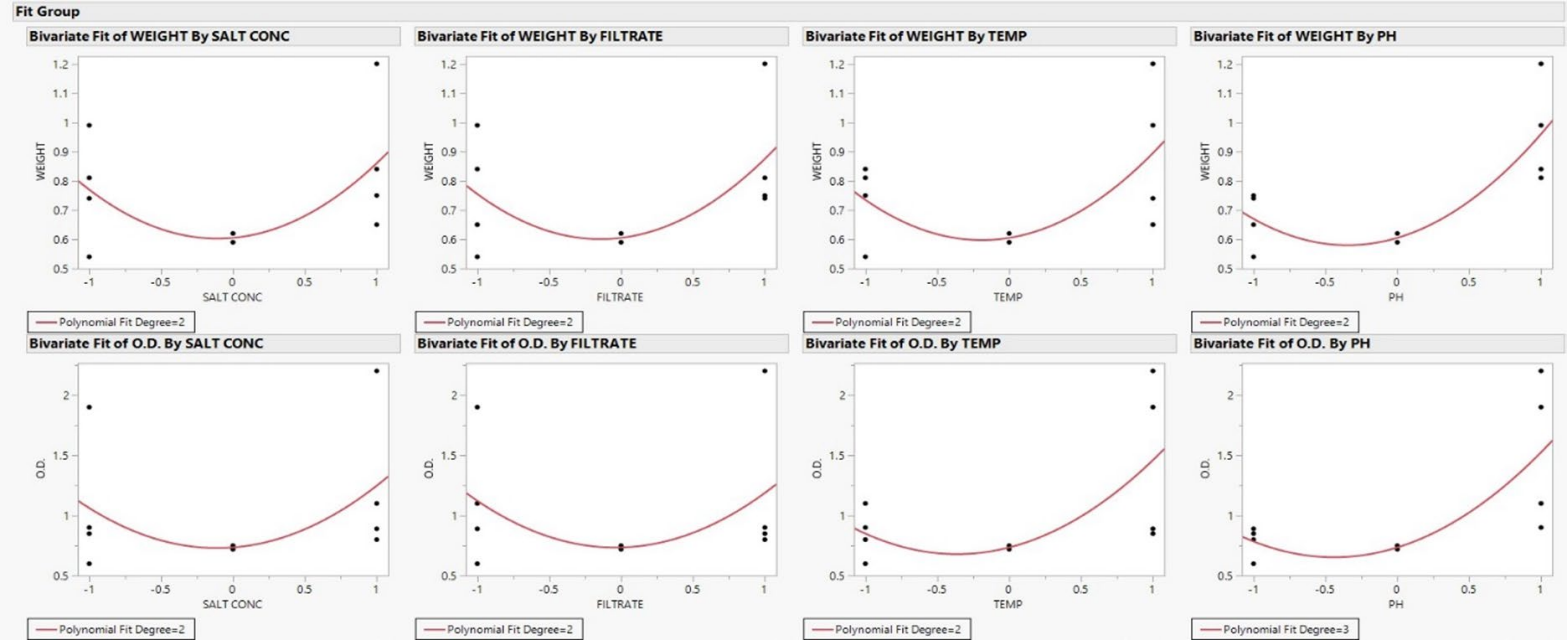
Bivariate fits of each response versus each factor. These plots provide a preliminary visual screening for strong effects and curvature.

In this work, the exploratory data analysis was used to find the most significant and correlations among the variables tested (Fig. 1). The Bivariate Fit graphs showed that the responses (weight and optical density, O.D.) were affected by temperature, pH, filtrate, and salt content. Such visual inspections are crucial for nanoparticle optimization because they help identify curvature, outliers, and dominant effects before statistical modeling. A strong positive linear relationship between pH and both responses indicated that an increase in pH values stimulated nanoparticle synthesis and stability. These results agree with Ahmed et al^29^., who showed that alkaline environments encourage the creation and stability of metal oxide nanoparticles.

The temperature exhibited a “J”-shaped relationship, this mean that at higher levels of temperature, more nanoparticles were produced. This is in agreement with research showing that high temperatures stimulate particle nucleation and proliferation^30^. The experimental design matrix and observed responses are presented in Table 2 and 3 while the regression results for weight and O.D. are summarized in Table 4. The regression analysis showed that pH was the only factor that had a statistically significant effect on weight (*p* = 0.0252; R_2_ = 0.683). However, both temperature and pH had a significant effect on O.D. and their interaction (R_2_ = 0.858, *p* < 0.05). A simple linear model did not work because of the curvature and the very low lack-of-fit test for O.D. (*p* = 0.0099). This suggests that pH and temperature interact in a way that is not linear, which is a common trend when optimizing nanoparticle synthesis^31^.

Overall, our results showed that temperature is the most important factor that affects optical properties, while pH is the most important factor that affects weight and O.D. The filtrate and salt concentration, on the other hand, didn’t add much, which means they can be lowered to save money when scaling up. So, the best way to get more nanoparticles is to keep the pH level alkaline and the temperature conditions as good as they can be. To get better control and repeatability, it is best to use RSM designs like Central Composite Design (CCD) to fine-tune process parameters and see quadratic effects^32^.

1 $$Y = \beta_{0}+ \beta_{1}X_{1} + \beta\:_{2}X_{2} + \beta_{11}X^{2} + \beta_{22}X^{2} + \beta_{12}X_{1}X_{2}$$

**Table 2:** Experimental design matrix and observed responses.

Pattern	Salt Conc	Filtrate	Temp	pH	Weight	O.D.
—-	−1	−1	−1	−1	0.54	0.6
–++	−1	−1	1	1	0.99	1.9
-+-+	−1	1	−1	1	0.81	0.9
-++-	−1	1	1	−1	0.74	0.85
+–+	1	−1	−1	1	0.84	1.1
+-+-	1	−1	1	−1	0.65	0.89
++–	1	1	−1	−1	0.75	0.8
++++	1	1	1	1	1.2	2.2
0000	0	0	0	0	0.62	0.72
0000	0	0	0	0	0.59	0.75

The complete 10-run design matrix and the observed responses are shown in Table 2.

**Table 3:** Parameter Estimates for the O.D. Model and Weight Model.

Term	Parameter Estimates For								
	O.D Model					Weight Model.			
	**Intercept**	**Temp**	**pH**	Temp*pH	**Intercept**		**Temp**	**pH**	**Filtrate**
Estimate	1.071	0.305	0.370	0.220	0.773		0.080	0.145	0.060
Std Error	0.078368	0.087618	0.087618	0.087618	0.043782		0.048949	0.048949	0.048949
t Ratio	13.67	3.48	4.22	2.51	17.66		1.63	2.96	1.23
Prob > |T|	*<.*0001*	0.0131*	0.0055*	0.0458*	*<.*0001*		0.1533	0.0252*	0.2662

Statistical power is the probability of correctly detecting a true effect.

### **Characterization of CuO-NPs and the as-prepared bio-nanocomposite coating**

**Fig. 2:**
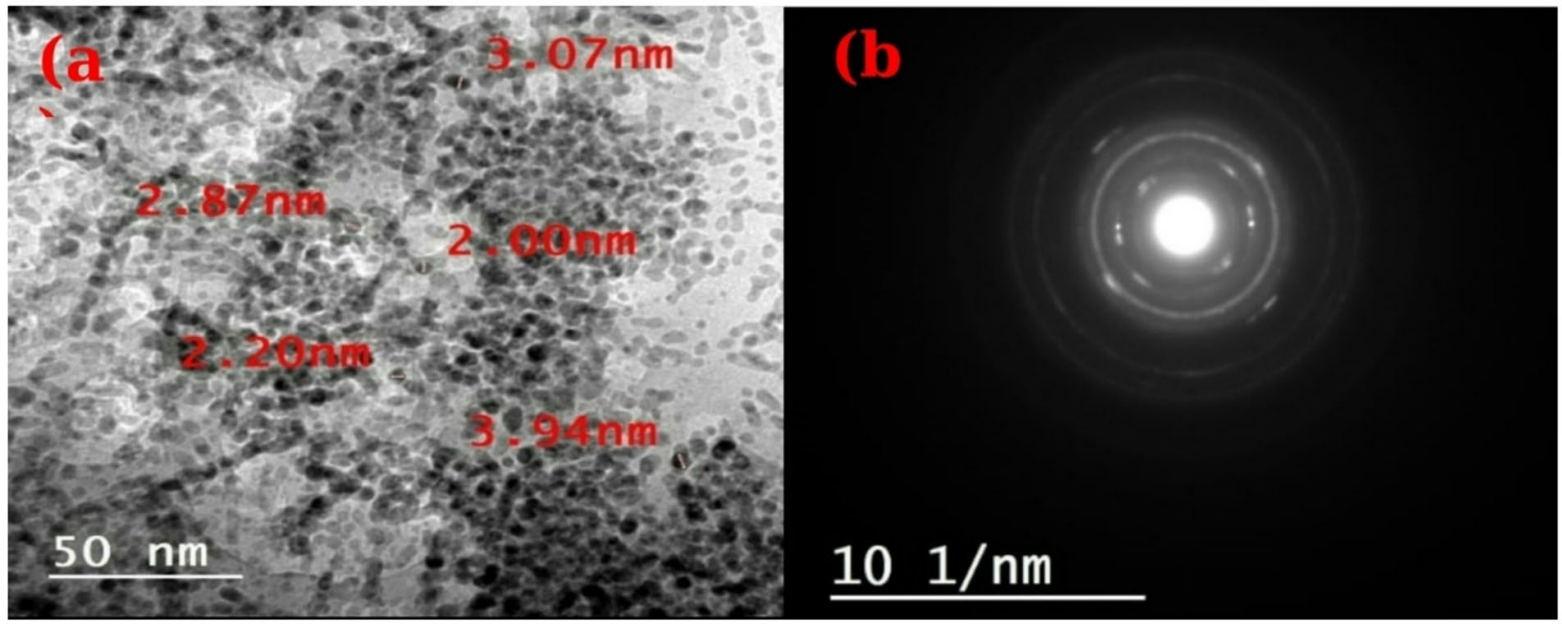
TEM (**a**) and SAED (**b**) of as-prepared CuO-NPs.

In Fig. 2a, CuO-NPs were detected with spherical forms successfully produced by the *Bacillus Safensis* FO-36b (NR_113945.1) strain. The as-prepared CuO-NPs had an average size in the range of 2.2–3.9 nm. The selected-area electron diffraction (SAED) patterns of the CuO-NPs are displayed in Fig. 2b. The poly-crystalline structure of the CuO-NPs was revealed by the good, crisp rings shown by the SAED pattern. The existence of CuO-NPs is indicated by a set of five visible diffraction rings that correspond to the (110), (002), (112−), (202), and (022) sets of planes. The size of CuO-NPs biosynthesized by fungi differs significantly from earlier research. According to Saravanakumar et al^33^., *Trichoderma asperellum* produced CuO-NPs with particle sizes ranging from 10 to 190 nm, which were nearly spherical.

**Fig. 3:**
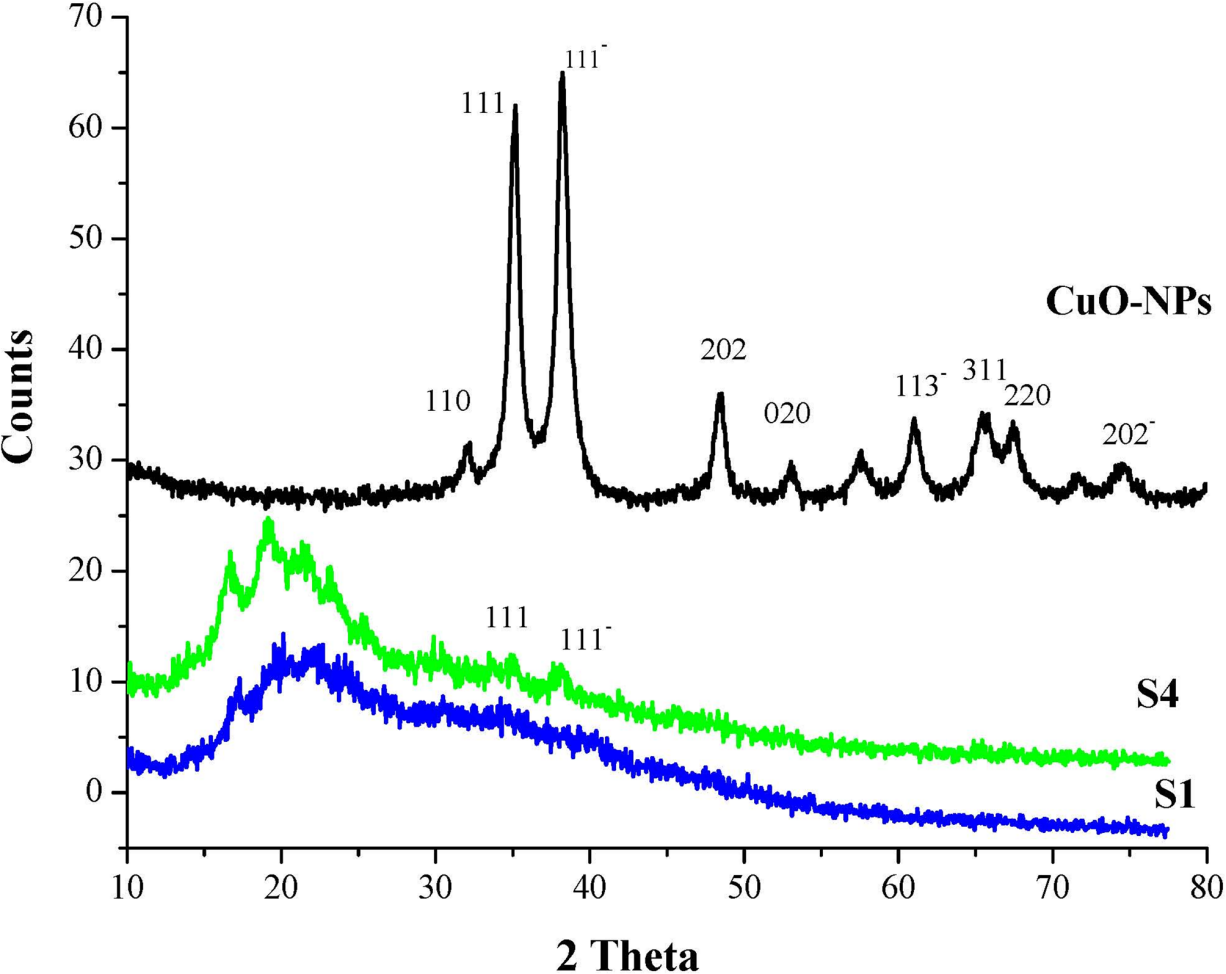
XRD diagrams of CuO-NPs, S1, and S4.

Figure 3 shows the XRD pattern of the CuO-NPs as well as the as-prepared bio-nanocomposite coating S1 and S4. The XRD pattern of the CuO-NPs shows that all the peaks correspond to the monoclinic structure of copper oxide and high crystallinity. X-ray diffraction analysis reveals the presence of several prominent peaks at 2θ equal 32.2°, 35.2°, 38.4°, 48.7°, 53.1°, 61.1°, 65.8°, 67.6° and 72.6° That are accurately match the standard monoclinic CuO reference pattern (JCPDS 45–0937), corresponding to the (110), (111), (111¯), (202), (020), (113¯), (311), (220) and (202¯) crystallographic planes, respectively^34,35^. The diffractogram for S1 shows broad peaks at 2θ = 22.9° as a result of the amorphous nature of St and PVA. Additionally, the XRD pattern of bio-nanocomposite coating S4 showed a broad peak at 2θ = 22.9°as well as unique signals of CuO-NPs with low intensity at 2θ = 35.2° and 38.4° that correspond to CuO-NPs crystallographic plane (111) and (111¯), respectively.

**Fig. 4:**
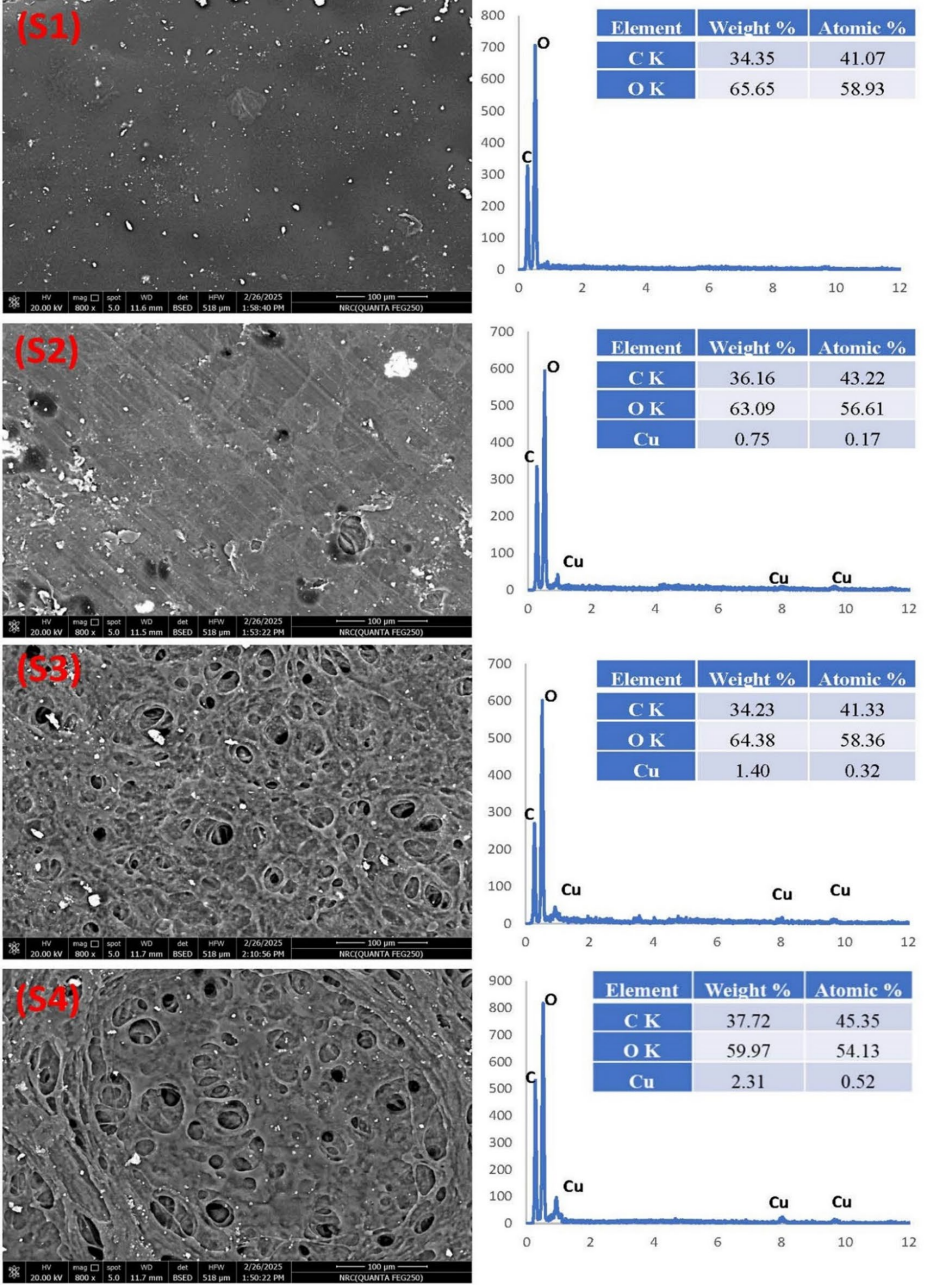
SEM characteristics of as-prepared films and their corresponding EDX.

The morphological surface structure of the bio-nanocomposite film was examined using SEM (Fig. 4). The dried polymer pieces were first positioned on Cu grade and then covered with gold before being placed into the SEM apparatus. Because of the hydrogen bonds that form between St and PVA, the St/PVA film (S1) has a homogeneous and compatible appearance and a considerably smooth surface. However, the CuO-NPs in St/PVA aid in changing the shape of the produced bio-nanocomposite film S2 into a rough surface with some holes, and these holes increase with increasing content of CuO-NPs in S3 and S4 films. Additionally, the EDX is used to confirm the presence of CuO-NPs in the bio-nanocomposite film. The EDX profile of S1 showed two peaks at 0.23 and 0.5 keV, corresponding to the K-shell electrons of carbon and oxygen atoms. The inset **Table** in Fig. 4 shows that the weight% of carbon and oxygen is 34.35% and 65.65%, respectively. The other samples (S2, S3, and S4) show additionally peaks at energies 1.0, 8.0, and 8.9 keV corresponding to CuO-NPs. The inserted **Table** in Fig. 4 showed that the weight% of Cu for samples S2, S3, and S4 is 0.75%, 1.40%, and 2.31%, respectively, which increases with increasing content of CuO-NPs in the as-prepared films.

**Fig. 5:**
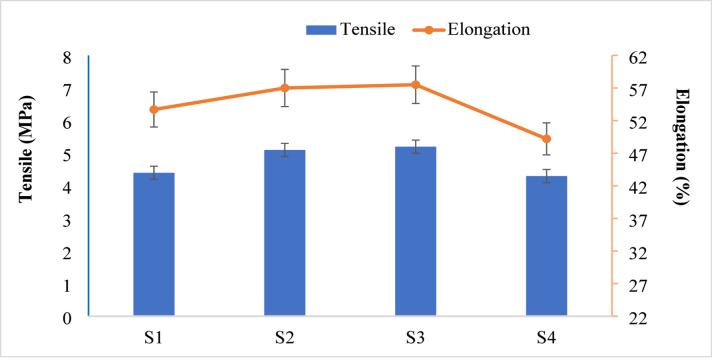
The mechanical properties of the prepared bio-nanocomposite films.

The mechanical characteristics of the produced bio-nanocomposite films based on St and PVA are significantly impacted by the inclusion of CuO-NPs. The data in Fig. 5 show that elongation at break (%) and tensile strength (MPa) change as the content of NPs varies. The bio-nanocomposite film S1, which has no presence of Cu-NPs, has a tensile strength of 4.4 MPa and an elongation at break of 53.7%. Whereas, the CuO-NPs at low contents of 20 and 40 mg enhance the tensile strength to 5.1 and 5.2 MPa and an elongation at break to 57 and 57.5% for bio-nanocomposite films S2 and S3, respectively. This enhancement can be attributed to the uniform dispersion of CuO-NPs and their strong interfacial interactions with hydroxyl groups of starch and PVA, which facilitate effective stress transfer and limit excessive polymer chain mobility without causing brittleness^36^. While the highest content of CuO-NPs (80 mg) in the bio-nanocomposite film S4 causes a decrease in both tensile strength (4.3 MPa) and elongation (49.2%). This phenomenon is associated with partial NPs agglomeration at higher filler contents, which breaks the continuity of the polymer matrix and causes localized stress concentration points. Under tensile loading, these agglomerates obstruct efficient stress distribution, which lowers mechanical performance^37,38^.

Table 4 shows the WVTR and OTR of the prepared St/PVA/CuO-NPs bio-nanocomposite films. The control sample S1, which was synthesized from St and PVA (4:1) without CuO-NPs, exhibits the highest WVTR (1809 g/m^2^.day) and the lowest OTR (0.08 cc/m^2^.day), which indicates a high diffusion of water vapor and sufficient resistance to oxygen diffusion. As the Cu-NPs content increases, the WVTR decreases, and the OTR increases considerably. In general, the incorporation of CuO-NPs in the polymer matrex demonstrating enhanced water vapor resistance and controlled oxygen permeation, ideal for cheese preservation.

Although SEM images (Fig. 4) show micron-scale surface pores, these features are not always continuous or through-thickness channels. Barrier properties are largely controlled by the bulk diffusion path, materials’ hydrophobicity, polymer chain packing, and interfacial interactions^39,40^. The data showed that OTR increased from 0.08 cc/m2.day (S1) to 3.82 cc/m2.day (S4) as shown in Table 4, which might be because of the formation of the micron-scale surface pores. Despite these microstructural characteristics, the WVTR decreased from 1809 g/m²·day (S1) to 1410 g/m²·day (S3). This could be because CuO-NPs create a zigzag diffusion pathway that greatly increases the water vapor’s travel distance and can form hydrogen bonds with water molecules^41^.

**Table 4:** The permeability of the as-prepared bio-nanocomposite films.

Sample	WVTR (g/m^2^.day)	OTR (cc/m^2^.day)
S1	1809 ± 25	0.08 ± 0.01
S2	1767 ± 20	1.10 ± 0.09
S3	1410 ± 19	2.51 ± 0.1
S4	1661 ± 22	3.82 ± 0.22

### **Antimicrobial activity of CuO-NPs and the as-prepared bio-nanocomposite coating**

**Fig. 6:**
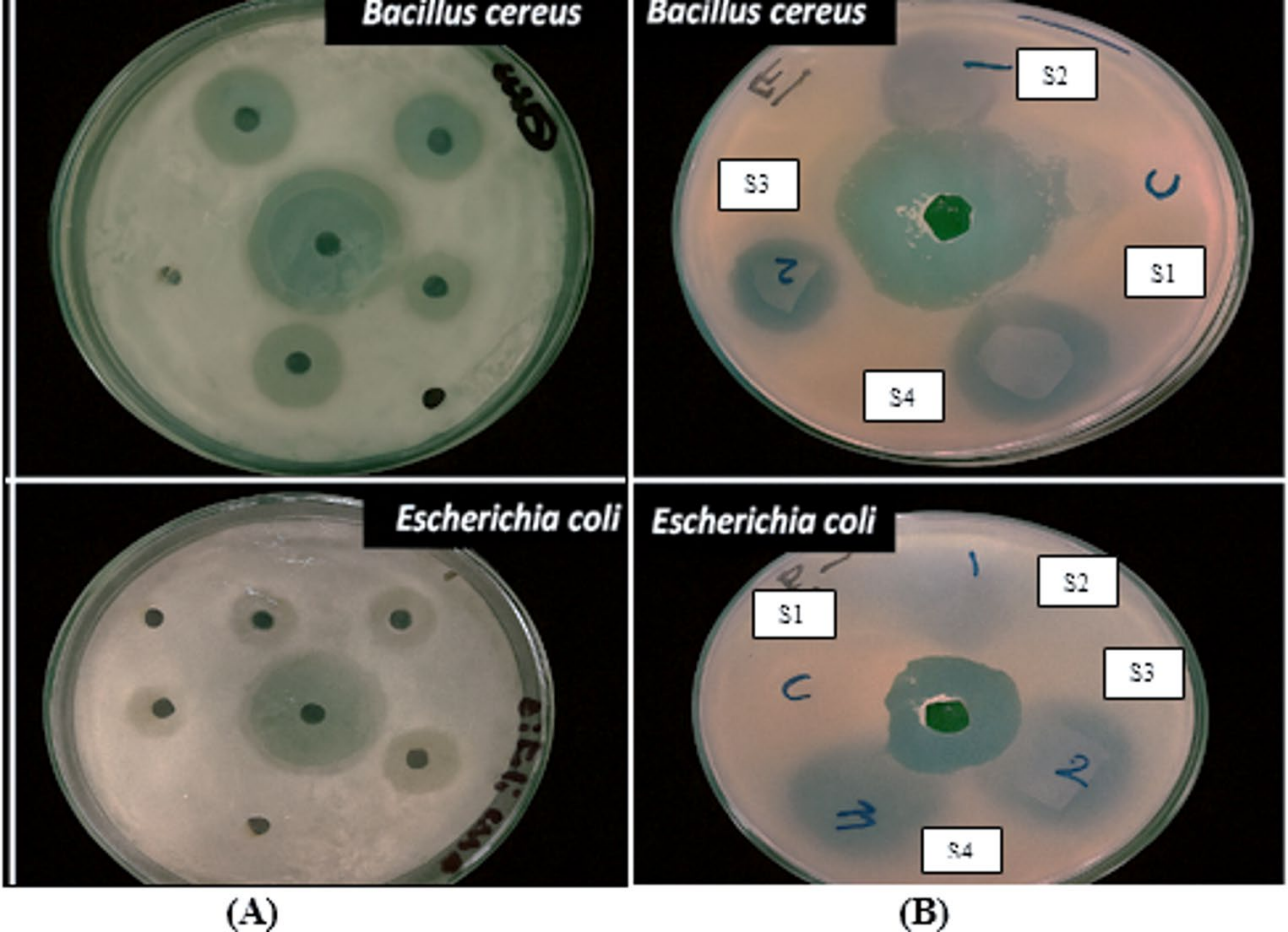
Antimicrobial activity of CuO-NPs (**A**) and bio-nanocomposite coating (**B**).

Antibacterial properties are essential for preventing the growth of bacteria in food packaging. We examined the antimicrobial efficacy of bio-nanocomposite films with varying CuO-NPs (0.0, 0.02, 0.04, and 0.08 g) added to obtain bio-nanocomposite coating S1, S2, S3, and S4, respectively. CuO-NPs and bio-nanocomposite films were tested for their antibacterial properties against *Bacillus cereus* ATCC 6629 and *Escherichia coli ATCC* 25,922 as food indicator bacteria. CuO-NPs demonstrated moderate antibacterial activity, as seen in Table 5; Fig. 6A, with maximum inhibition zones of 2.2 cm for *B. cereus* and 2.5 cm for *E. coli* at 40 mg/ml. On the other hand, the bio-nanocomposite film (Table 5; Fig. 6B) showed improved efficacy, attaining 2.9 cm and 2.5 cm inhibitory zones for *B. cereus* and *E. coli*, respectively, at just S4. According to Kumar et al^42^., these findings imply that the bio-nanocomposite matrix may improve the stability and bioavailability of CuO-NPs, potentially by fostering synergistic interactions between the NPs and the polymeric support, which would increase the antibacterial potency.

Additionally, *B. cereus* showed somewhat greater sensitivity than *E. coli*, which is in line with earlier research showing that, as a result of variations in cell wall construction, Gram-positive bacteria are often more vulnerable to metal oxide NPs^43^.

**Table 5:** Antimicrobial activity of CuO-NPs and the as-prepared bio-nanocomposite coating.

Tested bacteria	Treatment	Concentration (mg/ml)	Inhibition Zone (cm)	Standard Antibiotic
*dc Escherichia coli* ATCC 25,922	CuO-NPs	10	1.5	4
		20	1.5	
		30	1.8	
		40	2.2	
	bio-nanocomposite coating	S1	-ve	2.5
		S2	1.5	
		S3	2.1	
		S4	2.5	
*Bacillus cereus* ATCC 6629	CuO-NPs	10	1.6	3.5
		20	1.8	
		30	2	
		40	2.5	
	bio-nanocomposite coating	S1	-ve	3.2
		S2	1.8	
		S3	2.3	
		S4	2.9	

### The chemical composition of fresh UF- soft cheese

Table 6 explains the chemical composition of the prepared fresh ultra-filtered soft cheese before coating. The cheese has a high moisture content of 72.55%, which is typical for soft cheeses and affects its shelf life. The total solids are 27.45%, with a balanced composition of fat, protein, ash, and carbohydrates. The protein percentage of 9.43% is moderate, the fat content of 9.65% is significant for flavor, and the ash content is 2.62%, reflecting the cheese’s mineral content. The carbohydrate level is low, typical for cheese.

**Table 6:** The chemical composition of fresh UF- soft cheese treatments.

Sample	Moisture %	T.S %	Protein %	Fat %	Ash %	Carbohydrate %
UF- soft cheese	72.55 ± 0.05	27.45 ± 0.04	9.43 ± 0.01	9.65 ± 0.03	2.62 ± 0.25	5.75 ± 0.04

Data expressed as the average of three replicates ± SD.

### Change in total solids and pH values of different soft cheese samples

Table 7 presents the data on the impact of St/PVA/CuO-NPs bio-nanocomposites on total solids and the pH levels of UF soft cheese over various storage periods. The total solids in the control group increase slightly from 27.45% to 28.98% over 60 days, which could be attributed to moisture loss as the cheese matures and dries out. The addition of different coating materials results in a significant *P* < 0.05 reduction in total solids, particularly notable at higher concentrations of CuO-NPs. For example, cheese treatments with a coating of 0.08% CuO-NPs (S4) show a decrease from 27.45% to 25.73% over the storage period. This reduction may indicate that the bio-nanocomposite coating is affecting moisture retention, potentially leading to a higher moisture product compared to the control.

Furthermore, the control sample shows a gradually significant decrease (*P* < 0.05) in pH from 6.53 (fresh) to 6.28 (60 days). This decline indicates normal fermentation processes and microbial activity over time. The cheese coated with bio-nanocomposite coating S1 generally exhibits a more pronounced decrease in pH compared to the control. For instance, the cheese coating with S4 drops from 6.53 to 5.86 over 60 days, indicating that the coating by bio-nanocomposites blended with Cu-NPs may enhance acidity development. Interestingly, the pH levels significantly decrease (*P* < 0.05) at 15 days for some samples that are coated with S2 and S3 before stabilizing and slightly decreasing again, suggesting possible interactions between the bio-nanocomposite coating and the cheese matrix. This may possibly be due to Cu-NPs, which may improve the acidification process due to their antibacterial characteristics. Cu-NPs have excellent antibacterial characteristics, which can limit certain bacteria and pathogens^44^. Cu-NPs can improve fermentation processes by regulating microbial populations, which frequently results in increased synthesis of organic acids. Furthermore, moisture content affects the pH of cheese samples. Higher moisture levels can induce a lower pH (increased acidity) because moisture promotes microbial activity^45^. Bacteria metabolize lactose and generate acids, which contribute to the cheese’s overall acidity. Also, during cheese storage, proteins and fats are broken down to produce some of the organic acids^46^. The findings suggest that lower concentrations of Cu-NPs in bio-nanocomposite coating may be more suited to preserving product stability over time, while larger concentrations correspond with significant declines in total solids and pH. CuO-NPs antibacterial capabilities may extend shelf life; however, ensuring safety and quality requires balancing moisture loss and microbiological stability^47^.

**Table 7:** Change in total solids and pH values of uncoated and coated soft cheese as affected by St/PVA/Cu-NPs bio-nanocomposites and storage period*.

Chemical changes	Storage period (days)	Uncoated	St/PVA/Cu-NPs bio-nanocomposites			
		Control	S1	S2	S3	S4
Total solids %	**Fresh**	27.45^Da^ ±0.05	27.45^Aa^ ±0.05	27.45^Aa^ ±0.05	27.45^Aa^ ±0.05	27.45^Aa^ ±0.05
	**15**	27.91^Ca^ ±0.06	25.95^Cb^ ±0.02	25.52 ^Cb^±0.02	25.26^Cbc^±0.03	24.61^Cc^±0.05
	**30**	28.73^Bc^ ±0.05	26.18^BCb^±0.09	26.11^Bb^±0.05	25.29^Cc^±0.03	25.19^Cc^±0.04
	**45**	28.91^Aa^±0.06	26.36^Bb^±0.05	26.16^Bb^±0.06	25.76^Bc^±0.06	25.54^Bc^±0.07
	**60**	28.98^Aa^±0.03	26.45^Bb^ ±0.07	26.28^Bb^±0.08	25.95^Bc^±0.09	25.73^Bc^±0.04
pH	**Fresh**	6.53^Aa^ ±0.14	6.53^Aa^ ±0.14	6.53^Aa^ ±0.14	6.53^Aa^ ±0.14	6.53^Aa^ ±0.14
	**15**	6.49^Ba^ ±0.13	6.28^Bb^ ±0.11	6.13^Bc^ ±0.13	6.14^Bc^ ±0.16	6.15^Bc^ ±0.10
	**30**	6.41^Ca^ ±0.12	6.21^Cb^ ±0.15	6.11^Bc^ ±0.15	6.08^Bc^ ±0.15	6.10^Bc^ ±0.11
	**45**	6.36^Da^ ±0.17	6.14^Db^ ±0.12	6.12^Bb^ ±0.11	6.02^Bbc^ ±0.13	5.88^Cc^ ±0.12
	**60**	6.28^Ea^ ±0.11	6.12^Db^ ±0.13	6.11^Bb^ ±0.13	5.96^BCbc^ ±0.17	5.86^Cc^ ±0.19

Data expressed as mean of 3 replicates ± standard error. *Means with the same lower-case superscript (effect of coating) in the same row and upper case (effect of storage) in the same column are not significantly different (*P* > 0.05).

### Microbiological load of uncoated and coated soft cheese samples during the storage period

Table 8 showed the initial total bacterial counts for soft cheese ranged between 2.11 and 2.30 log CFU/g. The number of total bacterial counts increased with storage period in uncoated samples and samples coated with bio-nanocomposite coating S1. However, total bacterial counts for samples coated with bio-nanocomposite coating S2, S3, and S4 remained stable in the same log cycles for 30 days of storage. Also, a significant difference was observed between uncoated soft cheese (5.49 log CFU/g), coated cheese with S1 (4.80 log CFU/g), and soft cheese coated with S2, S3, and S4 (3.16, 2.49, and 2.19 log CFU/g, respectively) at 45 days of storage. Moreover, at 60 days of storage, total bacterial counts were 7.88 and 5.60 log CFU/g, respectively, for samples of uncoated soft cheese (control) and coated with S1 (blank). In contrast, the total bacterial counts for the coated cheese samples with bio-nanocomposite coating S2, S3, and S4 reached to 4.20, 3.40, and 3.00 log CFU/g, respectively, at the same storage time (60 days), indicating that added Cu-NPs was reduced microbial counts, which related to their antimicrobial activity^48^. Incorporating Cu-NPs enhanced the shelf life of soft cheese for 60 days of cold storage, and this effect reflects its antimicrobial activity, as confirmed in the present findings and other researchers^49,50^. The data also showed there were no significant differences between microbial counts for coated cheese with S3 or S4 during storage time.

The counts of mold and yeast were not detected initially or after 15 days of storage for all samples (Table 8). Some colonies were detected in control and samples coated with S1 (blank) at day 30, which indicated mold and yeast counts of 2.34 and 2.00 log CFU/g, respectively, for control and blank. At day 60 of storage, colony count increased to 4.37 and 3.10 log CFU/g, respectively, for the same samples. Nonetheless, the minor counts were detected for coated cheese with S2, which were first counted at day 45 (1.50 log CFU/g). The mold and yeast count for the coated cheese samples with S3 and S4 were initially detected at day 60 with counts of 1.73 and 1.00 log CFU/g, respectively, without significant differences between these samples. So, the antimicrobial activity of the film was directly related to the concentration of the CuO-NPs^51^.

As well, the coating and storage times had a significant effect on psychrotrophic counts in soft cheese (Table 8). Initial psychrotrophic count was detected at day 15 for the uncoated sample (control) with a count of 2.07 log CFU/g and increased with storage time to reach 6.45 log CFU/g at day 60 of storage. The psychrotrophic counts were detected for coated cheese samples with S1 (blank) and S2 at day 30 of storage (2.80 and 1.77 log CFU/g, respectively), and these numbers increased with storage time. The initial counts were detected for samples coated with S3 and S4 at day 45 with 1.14 and 1.00 log CFU/g, respectively. Also, the lowest numbers of psychrotrophic bacteria were observed in samples coated with S3 and S4, with counts of 2.00 and 1.82 log CFU/g, respectively, at day 60 of storage. Dissimilarity, uncoated cheese reported 6.45 log CFU/g at 60 days. Coating with St/PVA/Cu-NPs expanded preservation of soft cheese due to the antimicrobial properties^52^.

Generally, CuO-NPs’ positive charge may cause them to bind to bacterial cell walls. Also, CuO-NPs join up with carboxyl and amine groups found on microbial cell surfaces. As a result, bacteria that have more of these ionic groups on their cell surfaces are more susceptible to CuO-NPs and have a higher affinity for them. Cu is a structural component of several enzymes in living microorganisms. Therefore, High concentrations of Cu^2+^ ions generate harmful effects on microbial pathogens, causing ROS production and DNA intercalation. This generation via CuO-NPs, rather than soluble copper ions, is the primary factor in antibacterial mechanisms. On the other hand, microbes may extract toxic Cu^2+^ ions by solubilizing CuO-NPs, leading to increased cell permeability and unchecked passage of CuO-NPs, ultimately causing cell death^51,53^. The effectiveness of this approach increases with small nanometric levels of CuO-NPs due to their larger surface-to-volume ratio, increased ROS production per unit weight, and increased cell membrane penetration^44,54^. Finally, the counts of coliforms and spore-forming bacteria were not detected for all uncoated and coated cheese samples during the storage period. These are related to the hygienic roles and heated treatments for the milk that are applied during the soft cheese preparation. Also, the action of antimicrobial activates for the coated samples gave more preservative influence for cheese.

**Table 8:** Microbiological load of uncoated and coated soft cheese samples during storage period.

Microbial counts	Storage period (days)	Uncoated	St/PVA/Cu-NPs bio-nanocomposites			
		Control	S1	S2	S3	S4
Total bacterial counts	**Fresh**	2.30^Ae^±0.030	2.33^Ae^±0.042	2.18^Ac^±0.030	2.11^Ac^±0.033	2.14^Ab^±0.030
	**15**	3.47^Ad^±0.044	2.95^Bd^±0.011	2.50^Bc^±0.020	2.17^Cc^±0.030	2.11^Cb^±0.022
	**30**	4.53^Ac^±0.050	3.30^Bc^±0.040	2.82^Cc^±0.044	2.36^Cc^±0.033	2.00^Db^±0.020
	**45**	5.49^Ab^±0.044	4.80^Bb^±0.028	3.16^Cb^±0.033	2.49^Db^±0.052	2.19^Db^±0.033
	**60**	7.88^Aa^±0.30	5.60^Ba^±0.050	4.20^Ca^±0.042	3.40^Da^±0.040	3.00^Da^±0.035
Mold and yeast counts	**Fresh**	N.D ± 0.00	N.D ± 0.00	N.D ± 0.00	N.D ± 0.00	N.D ± 0.00
	**15**	N.D ± 0.00	N.D ± 0.00	N.D ± 0.00	N.D ± 0.00	N.D ± 0.00
	**30**	2.34^Ac^±0.011	2.00^Bc^±0.020	N.D ± 0.00	N.D ± 0.00	N.D ± 0.00
	**45**	3.24^Ab^±0.033	2.55^Bb^±0.025	1.50^Cb^±0.020	N.D ± 0.00	N.D ± 0.00
	**60**	4.73^Aa^±0.038	3.10^Ba^±0.030	2.00^Ca^±0.028	1.73^Da^±0.022	1.00^Da^±0.018
Psychotrophic counts	**Fresh**	N.D ± 0.00	N.D ± 0.00	N.D ± 0.00	N.D ± 0.00	N.D ± 0.00
	**15**	2.07^Ad^±0.020	N.D ± 0.00	N.D ± 0.00	N.D ± 0.00	N.D ± 0.00
	**30**	3.68^Ac^±0.033	2.80^Bc^±0.030	1.77^Cc^±0.025	N.D ± 0.00	N.D ± 0.00
	**45**	5.00^Ab^±0.040	3.28^Bb^±0.033	2.00^Cb^±0.028	1.14^Db^±0.020	1.00^Db^±0.015
	**60**	6.45^Aa^±0.038	4.90^Ba^±0.025	3.77Ca ± 0.03	2.00^Da^±0.027	1.82^Ea^±0.020

Means in the same row with different capital superscript letters are significantly different at *p* ≤ 0.05. Means in the same column with different small superscript letters are significantly different at *p* ≤ 0.05. ± SD.

## Conclusion

In the present study, CuO-NPs were synthesized in an eco-friendly process by *Bacillus Safensis* FO-36b (NR_113945.1). The characterization of CuO-NPs showed the formation of the poly-crystalline structure of the CuO-NPs. The used model can accurately map the curved response surface and predict the true optimal operating conditions. This sequential approach, screening to find key factors, then optimizing with RSM, is a hallmark of efficient scientific investigation. The fresh UF soft cheese has a characteristic chemical composition, with high moisture content and appropriate nutritional values. The analysis of pH and total solids variations in cheese as impacted by St/PVA/Cu-NPs bio-nanocomposites indicates complicated interactions that have a major impact on cheese quality. Coating with St/PVA/Cu-NPs expanded the preservation of soft cheese due to the antimicrobial properties. The antibacterial activity of the film was proportional to the concentration of Cu-NPs. Samples coated with S3 and S4 contained the fewest psychrotrophic bacteria, while untreated cheese had 6.45 log CFU/g after 60 days. The mold and yeast count for the coated cheese samples with S3 and S4 was initially detected at day 60.

Even though the nanocomposite materials offer antimicrobial and antioxidant functions, improved thermal stability, and extended food shelf life. However, the use of nanoparticles in food-contact materials is limited by nanoparticle or metal ion migration risks, potential toxicity, particle aggregation affecting optical and mechanical properties, and high costs.

## Data Availability

Data is provided within the manuscript or supplementary information files.

## References

[1] Fouda, A. et al. Eco-friendly approach utilizing green synthesized nanoparticles for paper conservation against microbes involved in biodeterioration of archaeological manuscript. *Int. Biodeterior. Biodegrad.* **142**, 160–169. 10.1016/j.ibiod.2019.05.012 (2019).

[2] Marooufpour, N., Alizadeh, M., Hatami, M. & Asgari Lajayer, B. in *Microbial Nanobionics: Volume 1, State-of-the-Art* (ed Ram Prasad) 63–85Springer International Publishing, (2019).

[3] Ali Abdulla, I., Gomaa, S. K. & Abd-elsalam, I. S. Biosynthesis of Biologically Active Silver Nano Particles Using Natural Red Pigment Under Optimized Conditions. *Egypt. J. Chem.* **68**, 395–401. 10.21608/ejchem.2025.350811.11122 (2025).

[4] Talebian, S., Shahnavaz, B., Nejabat, M., Abolhassani, Y. & Rassouli, F. B. Bacterial-mediated synthesis and characterization of copper oxide nanoparticles with antibacterial, antioxidant, and anticancer potentials. *Front. Bioeng. Biotechnol.* **11**, 1140010 (2023). 36949885 10.3389/fbioe.2023.1140010 PMC10025390

[5] Murugan, B. et al. Green synthesis of CuO nanoparticles for biological applications. *Inorg. Chem. Commun.* **155**, 111088. 10.1016/j.inoche.2023.111088 (2023).

[6] Abd El-Aziz, M. E. et al. Bio-nanocomposite edible coating based on chitosan, Arabic gum and CuO-NPs to improve the storage life of Hass avocado. *Food Saf. Risk*. **13**, 5. 10.1186/s40550-025-00123-z (2025).

[7] Alamer, H. A., Shawir, S. M. S., Kamel, R. M., Salama, A. M. & Sakr, H. Biodegradable films based on gum arabic, chitosan, and polyvinyl alcohol incorporating Hibiscus flower-derived carbon dots impact the postharvest quality of Barhi dates. *Int. J. Biol. Macromol.* **308**, 142723. 10.1016/j.ijbiomac.2025.142723 (2025). 40174849 10.1016/j.ijbiomac.2025.142723

[8] Abd El-Aziz, M. E., Morsi, S. M. M., Hasanin, M. S. & Youssef, A. M. Synthesis and characterization of cellulose/polylactic acid/MnO2 bio-nanocomposite as a promising coating of paper sheet for food packaging application. *Carbohydr. Polym. Technol. Appl.* **10**, 100789. 10.1016/j.carpta.2025.100789 (2025).

[9] Youssef, A. M., El-Aziz, A., Morsi, S. M. M. & M. E. & Development and evaluation of antimicrobial LDPE/TiO2 nanocomposites for food packaging applications. *Polym. Bull.*10.1007/s00289-022-04346-4 (2022).

[10] Liu, X. et al. Recent advances in chitosan-based active and intelligent packaging films incorporated with flavonoids. *Food Chemistry: X*. **25**, 102200. 10.1016/j.fochx.2025.102200 (2025). 39974528 10.1016/j.fochx.2025.102200 PMC11838128

[11] Markam, M., Chouksey, S. & Bajpai, A. in *Handbook of Nanofillers* (eds Shadpour Mallakpour & Chaudhery Mustansar Hussain) 1–36Springer Nature Singapore, (2024).

[12] Rashedy, A. A. et al. Arabic gum/chitosan/Zn–NPs composite film maintains the quality of Hass avocado fruit by delaying ripening and activating enzymatic defense mechanisms. *Sci. Rep.* **14**, 401. 10.1038/s41598-023-50642-y (2024). 38172333 10.1038/s41598-023-50642-y PMC10764304

[13] Agarwal, S., Singhal, S., Godiya, C. B. & Kumar, S. Prospects and Applications of Starch based Biopolymers. *Int. J. Environ. Anal. Chem.* **103**, 6907–6926. 10.1080/03067319.2021.1963717 (2023).

[14] Diyana, Z. N. et al. Physical Properties of Thermoplastic Starch Derived from Natural Resources and Its Blends: A Review. *Polymers* **13**, 1396. 10.3390/polym13091396 (2021). 33925897 10.3390/polym13091396 PMC8123420

[15] Abd El-Aziz, M. E. & Morsi, S. M. M. in *Polymer and Biopolymer Nanocomposites* (eds Muhammad Moniruzzaman & A. Vijaya Bhaskar Reddy) 29–47Woodhead Publishing, (2026).

[16] Sudheesh, C., Varsha, L., Siddiqui, S. A., Sunooj, K. V. & Pillai, S. Exploring urea as a prospective auxiliary for starch functionalization: A concise review on modified starch properties and the sustainable packaging films. *Food Chem.* **455**, 139914. 10.1016/j.foodchem.2024.139914 (2024). 38823124 10.1016/j.foodchem.2024.139914

[17] Mueller, E. et al. Development of ternary polymeric films based on cassava starch, pea flour and green banana flour for food packaging. *Int. J. Biol. Macromol.* **256**, 128436. 10.1016/j.ijbiomac.2023.128436 (2024). 38016616 10.1016/j.ijbiomac.2023.128436

[18] Ayoub, A. W., Sayed, S. M., Hefnawy, Y. A. & Youssef, A. M. Innovative use of chitosan /PVA/GE/ZnO biofilms based on Sage nanoemulsion for sustainable antimicrobial packaging of chilled chicken meat parts. *J. Food Meas. Charact.* **19**, 4961–4984. 10.1007/s11694-025-03306-6 (2025).

[19] Pham, T. L. & Nguyen, V. S. Polyvinyl alcohol/cellulose nanocrystals incorporated with apple waste-derived carbon dots as an excellent UV food packaging material. *Mater. Today Commun.* **41**, 111091. 10.1016/j.mtcomm.2024.111091 (2024).

[20] Youssef, A. M., El-Sayed, H. S., El-Sayed, S. M., Fouly, M. & Abd El-Aziz, M. E. Novel Bionanocomposites Based on Cinnamon Nanoemulsion and TiO2-NPs for Preserving Fresh Chicken Breast Fillets. *Food Bioprocess Technol.* **16**, 356–367. 10.1007/s11947-022-02934-w (2023).

[21] Nassar, A. R. A., Atta, H. M., Abdel-Rahman, M. A., Naghy, E., Fouda, A. & W. S. & Myco-synthesized copper oxide nanoparticles using harnessing metabolites of endophytic fungal strain Aspergillus terreus: an insight into antibacterial, anti-Candida, biocompatibility, anticancer, and antioxidant activities. *BMC Complement. Med. Ther.* **23**10.1186/s12906-023-04056-y (2023). 10.1186/s12906-023-04056-y PMC10363295 37481531

[22] Pretorius, J. C., Magama, S., Zietsman, P. C. & van Wyk, B. E. Growth inhibition of plant pathogenic bacteria and fungi by extracts from selected South African plant species. *South. Afr. J. Bot.* **69**, 186–192. 10.1016/S0254-6299(15)30344-6 (2003).

[23] El-Sayed, S. M. & El-Sayed, H. S. Incorporating white radish extract and L. plantarum to ultra-filtrated soft cheese for boost its functional, microbiological, and sensory qualities. *Biocatal. Agric. Biotechnol.* **58**, 103188. 10.1016/j.bcab.2024.103188 (2024).

[24] Association of Official Analytical, C. Official methods of analysis of the Association of Official Analytical Chemists. Vol. 11. The Association, (2000).

[25] Vanderzant, C. & Splittstoesser, D. F. Compendium of Methods for the Microbiological Examination of foods, Washington D. *DC: Am. Public. Health Association* (1992).

[26] Peng, C. Y. & J *Data analysis using SAS* (Sage, 2008).

[27] Alali, A., Hosseini-Abari, A., Bahrami, A. & Yazdan Mehr, M. Biosynthesis of Copper Oxide and Silver Nanoparticles by Bacillus Spores and Evaluation of the Feasibility of Their Use in Antimicrobial Paints. *Materials* **16**, 4670. 10.3390/ma16134670 (2023). 37444983 10.3390/ma16134670 PMC10342331

[28] Dolati, M., Tafvizi, F., Salehipour, M., Komeili Movahed, T. & Jafari, P. Biogenic copper oxide nanoparticles from Bacillus coagulans induced reactive oxygen species generation and apoptotic and anti-metastatic activities in breast cancer cells. *Sci. Rep.* **13**, 3256. 10.1038/s41598-023-30436-y (2023). 36828883 10.1038/s41598-023-30436-y PMC9958044

[29] Ahmed, S., Ahmad, M., Swami, B. L. & Ikram, S. A review on plants extract mediated synthesis of silver nanoparticles for antimicrobial applications: A green expertise. *J. Adv. Res.* **7**, 17–28. 10.1016/j.jare.2015.02.007 (2016). 26843966 10.1016/j.jare.2015.02.007 PMC4703479

[30] Kumar, V. & Yadav, S. K. Plant-mediated synthesis of silver and gold nanoparticles and their applications. *J. Chem. Technol. Biotechnol.* **84**, 151–157. 10.1002/jctb.2023 (2009).

[31] Gunst, R. F. Response Surface Methodology: Process and Product Optimization Using Designed Experiments. *Technometrics* **38**, 284–286. 10.1080/00401706.1996.10484509 (1996).

[32] Jensen, W. A. Response Surface Methodology: Process and Product Optimization Using Designed Experiments 4th edition. *J. Qual. Technol.* **49**, 186–188. 10.1080/00224065.2017.11917988 (2017).

[33] Saravanakumar, K. et al. Biosynthesis and characterization of copper oxide nanoparticles from indigenous fungi and its effect of photothermolysis on human lung carcinoma. *J. Photochem. Photobiol., B*. **190**, 103–109. 10.1016/j.jphotobiol.2018.11.017 (2019). 30508758 10.1016/j.jphotobiol.2018.11.017

[34] Mahmoud, N. M. R., Mohamed, H. I., Ahmed, S. B. & Akhtar, S. Efficient biosynthesis of CuO nanoparticles with potential cytotoxic activity. *Chem. Pap.* **74**, 2825–2835. 10.1007/s11696-020-01120-6 (2020).

[35] Nabila, M. I. & Kannabiran, K. Biosynthesis, characterization and antibacterial activity of copper oxide nanoparticles (CuO NPs) from actinomycetes. *Biocatal. Agric. Biotechnol.* **15**, 56–62. 10.1016/j.bcab.2018.05.011 (2018).

[36] Hasanin, M. S. & Youssef, A. M. Ecofriendly bioactive film doped CuO nanoparticles based biopolymers and reinforced by enzymatically modified nanocellulose fibers for active packaging applications. *Food Packaging Shelf Life*. **34**, 100979. 10.1016/j.fpsl.2022.100979 (2022).

[37] Moustafa, A. B., El-Aziz, A., Rabea, M. E., Essawy, H. A. & A. M. & Polystyrene-montmorillonite core–shell particles via Pickering emulsion polymerization and their use as reinforcing additives for polypropylene and ethylene vinyl acetate. *Polym. Eng. Sci.* **55**, 1546–1552. 10.1002/pen.24094 (2015).

[38] Guimarães, M., Botaro, V. R., Novack, K. M., Teixeira, F. G. & Tonoli, G. H. D. Starch/PVA-based nanocomposites reinforced with bamboo nanofibrils. *Ind. Crops Prod.* **70**, 72–83. 10.1016/j.indcrop.2015.03.014 (2015).

[39] Ashfaq, J. et al. Enhancement of Thermal and Gas Barrier Properties of Graphene-Based Nanocomposite Films. *ACS Omega*. **8**, 41054–41063. 10.1021/acsomega.3c02885 (2023). 37970029 10.1021/acsomega.3c02885 PMC10633891

[40] Abd El-Aziz, M. E. & Morsi, S. M. M. Metal nanoparticles in food packaging: Benefits, functions and limitations. *Food Control*. **183**, 111929. 10.1016/j.foodcont.2025.111929 (2026).

[41] Abdo, S. M., Youssef, A. M., El-Liethy, M. A. & Ali, G. H. Preparation of simple biodegradable, nontoxic, and antimicrobial PHB/PU/CuO bionanocomposites for safely use as bioplastic material packaging. *Biomass Convers. Biorefinery*. **14**, 28673–28683. 10.1007/s13399-022-03591-x (2024).

[42] Kumar, P., Huo, P., Zhang, R. & Liu, B. Antibacterial Properties of Graphene-Based Nanomaterials. *Nanomaterials* **9**, 737. 10.3390/nano9050737 (2019). 31086043 10.3390/nano9050737 PMC6567318

[43] Raghupathi, K. R., Koodali, R. T. & Manna, A. C. Size-Dependent Bacterial Growth Inhibition and Mechanism of Antibacterial Activity of Zinc Oxide Nanoparticles. *Langmuir* **27**, 4020–4028. 10.1021/la104825u (2011). 21401066 10.1021/la104825u

[44] Youssef, A. M. et al. Synthesis and evaluation of eco-friendly carboxymethyl cellulose/polyvinyl alcohol/CuO bionanocomposites and their use in coating processed cheese. *RSC Adv.* **10**, 37857–37870. 10.1039/D0RA07898K (2020). 35515154 10.1039/d0ra07898k PMC9057223

[45] Shah, N. P. & Ravula, R. R. Influence of water activity on fermentation, organic acids production and viability of yogurt and probiotic bacteria. *Australian J. Dairy. Technol.* **55**, 127 (2000).

[46] Murtaza, M. S. et al. Physicochemical, techno-functional, and proteolytic effects of various hydrocolloids as fat replacers in low-fat cheddar cheese. *Front. Sustainable Food Syst.* **8**, 1440310. 10.3389/fsufs.2024.1440310 (2024).

[47] Jadhav, R. et al. An overview of antimicrobial nanoparticles for food preservation. *Mater. Today: Proc.* **72**, 204–216. 10.1016/j.matpr.2022.07.045 (2023).

[48] Saravanakumar, K., Sathiyaseelan, A., Mariadoss, A. V. A., Xiaowen, H. & Wang, M. H. Physical and bioactivities of biopolymeric films incorporated with cellulose, sodium alginate and copper oxide nanoparticles for food packaging application. *Int. J. Biol. Macromol.* **153**, 207–214. 10.1016/j.ijbiomac.2020.02.250 (2020). 32105688 10.1016/j.ijbiomac.2020.02.250

[49] Tran, T. H. & Nguyen, V. T. Copper Oxide Nanomaterials Prepared by Solution Methods, Some Properties, and Potential Applications: A Brief Review. *International Scholarly Research Notices* 856592, (2014). 10.1155/2014/856592 (2014). 10.1155/2014/856592 PMC4897379 27437488

[50] Phutanon, N., Motina, K., Chang, Y. H. & Ummartyotin, S. Development of CuO particles onto bacterial cellulose sheets by forced hydrolysis: A synergistic approach for generating sheets with photocatalytic and antibiofouling properties. *Int. J. Biol. Macromol.* **136**, 1142–1152. 10.1016/j.ijbiomac.2019.06.168 (2019). 31247232 10.1016/j.ijbiomac.2019.06.168

[51] Huh, A. J. & Kwon, Y. J. Nanoantibiotics: A new paradigm for treating infectious diseases using nanomaterials in the antibiotics resistant era. *J. Controlled Release*. **156**, 128–145. 10.1016/j.jconrel.2011.07.002 (2011). 10.1016/j.jconrel.2011.07.002 21763369

[52] Xing, Y. et al. Antimicrobial Nanoparticles Incorporated in Edible Coatings and Films for the Preservation of Fruits and Vegetables. *Molecules* **24**, 1695. 10.3390/molecules24091695 (2019). 31052263 10.3390/molecules24091695 PMC6539459

[53] Youssef, A. et al. Development of bionanocomposite materials and its use in coating of Ras cheese. *Food Chem.* **270**, 467–475 (2019). 30174073 10.1016/j.foodchem.2018.07.114

[54] Karlsson, H. L. et al. Cell membrane damage and protein interaction induced by copper containing nanoparticles—Importance of the metal release process. *Toxicology* **313**, 59–69. 10.1016/j.tox.2013.07.012 (2013). 23891735 10.1016/j.tox.2013.07.012

