# Correction: Preparation of starch/PVA/Cu-NPs bio-nanocomposites in packing as preservation of UF soft cheese

**DOI:** 10.1038/s41598-026-54303-8

**Published:** 2026-05-21

**Authors:** Heba A. El-Refai, Sanaa K. Gomaa, Rania A. Zaki, Samah M. El-Sayed, Hoda S. El-Sayed, Youssef R. Hassan, Abdelmonem Elrefaey, M. E. Abd El-Aziz

**Affiliations:** 1 Chemistry of Natural and Microbial Products Department, National Research Centre, 33 El Bohouth St. (former El Tahrir St.), Dokki, P.O. 12622, Giza, Egypt; 2 Dairy Science Department, National Research Centre, 33 El Bohouth St. (former El Tahrir st.), Dokki, P.O. 12622, Giza, Egypt; 3 Packaging Materials Department, National Research Centre, 33 El Bohouth St. (former El Tahrir st.), Dokki, P.O. 12622, Giza, Egypt; 4 School of Computing and Augmented Intelligence, Arizona State University, Tempe, Arizona, AZ 85281 USA; 5 Polymers and Pigments Department, National Research Centre, 33 El Bohouth St., Dokki, P.O. 12622, Giza, Egypt

Correction to: *Scientific Reports* 10.1038/s41598-026-44328-4, published online 04 April 2026

In the original version of this Article, the name of the author Sanaa K. Gomaa was duplicated.

The original Article has been corrected.

